# Jumping to conclusions bias, psychosis and impulsivity in early stages of Parkinson’s disease

**DOI:** 10.1007/s00415-023-11904-x

**Published:** 2023-08-09

**Authors:** Ioanna Pachi, Vassilis Papadopoulos, Lida Alkisti Xenaki, Christos Koros, Athina Maria Simitsi, Anastasia Bougea, Maria Bozi, Nikos Papagiannakis, Rigas Filippos Soldatos, Dimitra Kolovou, George Pantes, Nikolaos Scarmeas, Georgios Paraskevas, Konstantinos Voumvourakis, Constantin Potagas, Sokratis G. Papageorgiou, Konstantinos Kollias, Nikos Stefanis, Leonidas Stefanis

**Affiliations:** 1 1st Department of Neurology, Aeginition Hospital, National and Kapodistrian University of Athens, 72-74, Vassilissis Sofias Av., 11528 Athens, Greece; 2 1st Department of Psychiatry, Aeginition Hospital, National and Kapodistrian University of Athens, 72-74 Vas. Sofias Av., Athens, Greece; 3 2nd Department of Neurology, Attikon University Hospital, National and Kapodistrian University of Athens, 1 Rimini Str., Athens, Greece; 4 Taub Institute for Research in Alzheimer’s Disease and the Aging Brain, Columbia University, New York, NY USA

**Keywords:** Jumping to conclusions, Psychosis, Early, Parkinson’s disease

## Abstract

**Objectives:**

The aim was to explore the correlations between Jumping to Conclusions (JtC) tendency and neuropsychiatric features in patients with early Parkinson’s disease (PD).

**Background:**

According to few reports, PD patients with impulsive–compulsive behaviors (ICBs) are prone to working memory difficulties including JtC bias. The correlation of psychotic features and JtC tendency remains still unclear.

**Methods:**

Healthy controls and patients within 3 years of PD onset were recruited. Participants were examined for psychotic symptoms using a 10 question PD-specific psychosis severity scale. JtC was measured by a probalistic reasoning scenario (beads task). In PD group, medication use, motor and non-motor symptoms were documented. Impulsivity was evaluated using the Questionnaire for Impulsive–Compulsive Disorders in PD (QUIP).

**Results:**

The prevalence of JtC bias was 9% (6/70) in healthy individuals, compared to 32% (22/68) of PD group [*p* = 0.001]. No association was detected between the presence of JtC tendency and PD-associated psychosis (*p* = 0.216). Patients with JtC had shorter duration of PD, more tremor-dominant PD subtype and higher QUIP scores, regardless of the dopaminergic therapy (*p* = 0.043, *p* = 0.015, *p* = 0.007, respectively). A trend towards attention and inhibition control deficit was noticed in JtC patients.

**Conclusions:**

We found a high prevalence of JtC bias in early, cognitively intact PD population and a potential link between subthreshold ICBs and poor performance on beads task. Additional studies are needed to confirm our results and elaborate on the mechanisms that correlate impulsivity with JtC tendency, which are likely to be different from those mediating psychotic features in early PD.

**Supplementary Information:**

The online version contains supplementary material available at 10.1007/s00415-023-11904-x.

## Introduction

The reasoning bias of jumping to conclusions (JtC) consists of a tendency to have an impaired decision process in which assumptions are made on limited data-gathering [1]. This style of information process has been suggested to favor the formation of abnormal inferences leading to the adoption of delusional beliefs. It has been studied in several psychotic states including individuals with delusions, at-risk psychotic groups and delusion-remitted subjects [2]. The underlying mechanisms for this cognitive bias are not yet clear, although some hypotheses involve emotional processes or neurocognitive deficits, such as working memory impairment [3].

Apart from schizophrenia-spectrum disorders, JtC bias and dysfunctions in decision-making are cardinal features in a range of mental disorders including addiction [4], eating and anxiety disorders [5, 6], as well as neurodegenerative disorders, such as Parkinson’s disease (PD). Especially, in untreated PD population, impairment in reward learning, novelty processing, visuospatial functions and verbal memory have been described [7, 8]. The beads task, a data-gathering paradigm indicating marked JtC tendency, has been referred to as “reflection impulsivity” measure in de novo PD [9] and has been linked to impulsive–compulsive behaviors (ICBs) in moderate PD stages [10]. It has been proposed that the use of dopaminergic agonists might facilitate the poor performance in beads task [11]. Interestingly, the presence of risk-taking tendencies has been noted in patients with mild disease severity [12], yet not in de novo PD patients [13]. Furthermore, global cognitive function has been found relatively intact in patients with PD during decision-making task, indicating that decision-making ability has a basis different from that of general intellectual ability, possibly implicating social and emotional cognition [14]. Despite the conflicting results, it seems that depressive features might not explain the risky decision-making patterns of PD patients [13]. Among non-motor manifestations, fatigue might be associated with impaired decision-making process [15]. The motor, non-motor and neuropsychiatric factors that contribute particularly to the presence of JtC bias in early PD population remain still unclear.

The aim of our study was to investigate the prevalence of JtC in healthy individuals and patients with early PD and explore the correlations between JtC tendency and clinical, neurocognitive and neuropsychiatric features. We hypothesized that patients in early period of PD would be more likely to jump to conclusions, compared to healthy individuals. We also speculated that psychotic symptoms and impulsive behaviors would be highly associated with the presence of JtC tendency in our PD sample.

## Methods

### Participants

The study had a cross-sectional design. The study population was recruited from the Movement Disorder Clinic in the 1st and 2nd Neurology Department in National and Kapodistrian University of Athens (NKUA). The Movement Disorder Society (MDS) Clinical Diagnostic Criteria for PD were implemented to confirm the diagnosis in patients of the sample. Inclusion criteria were age older than 18 years and up to 3-year duration of motor symptoms. Patients with clinical features suggestive of primary atypical Parkinsonism or diagnosis of dementia were excluded. Age-matched individuals with no symptoms of PD or dementia and no family history of PD were grouped as “healthy controls” and mainly involved spouses of patients with PD.

### Clinical assessments

Demographic characteristics were documented for both healthy controls and patients with PD. Data on education level and clinical characteristics such as family history of PD, duration of disease and age of symptom onset were recorded for the PD group. Information on treatment strategies including levodopa equivalent daily dose (LEDD) and use of specific drug categories (levodopa, dopaminergic agonists, and monoamine oxidase [MAO] inhibitors) was also documented. The method for converting the total daily dopaminergic therapeutic dose in LEDD was obtained from published formulas [16].

Motor signs and symptoms were evaluated using the Movement Disorder Society Unified Parkinson’s Disease Rating Scale (MDS-UPDRS) part II and III motor subscales [17] and the Hoehn and Yahr (HY) scale [18]. Individuals’ ability to function in activities of daily living was assessed by the Schwab and England Activities of Daily Living (SE-ADL) scale [19]. Patients were classified as having tremor-dominant (i.e., presenting marked resting tremor with mild bradykinesia or rigidity), akinetic-rigid (i.e., presenting marked akinesia or bradykinesia and rigidity with no or only mild tremor) or mixed phenotypes, depending on their essential motor manifestation.

Furthermore, non-motor features were measured using the MDS-UPDRS part I scale [17]. Other measures included the REM sleep behavior disorder screening questionnaire (RBDQ), with a screening cutoff of ≥ 5 indicating RBD [20], and the Sniffin’ Sticks Screening test assessing olfaction [21].

### Neuropsychiatric examination

Global cognitive abilities were assessed with the Montreal Cognitive Assessment (MoCA) and Mild Cognitive Impairment for patients with PD was defined at the recommended cutoff value of < 26 [22].

Frontal Assessment Battery (FAB) was implemented to examine the executive and frontal lobe dysfunction in patients with PD. A cut off score of ≤ 12 in FAB was optimal to indicate frontal impairment [23].

Depression was examined using the 15-item Geriatric Depression Scale (GDS) with a cutoff score of ≥ 5 indicating presence of clinically significant depressive features [24], while apathetic symptoms were tested using the 1.5 item of MDS-UPDRS scale part I with a cutoff score of ≥ 1 indicating presence of apathy [17].

Psychotic features were evaluated using an easy-to-administer 10-question PD-specific psychosis severity scale (10PDQ). This scale contains ten items. The first five questions identify the type of hallucination (visual, auditory, olfactory, sense of presence) or delusion, while the last five quantify the intensity, frequency, insight and impact of the worst psychotic experience in the life of the patient and the family. The range of score for each item is 0–4 and the total score adds all ten items (range: 0–40) [25]. Subjects were defined as “10PDQ cases” when they had a total score > 0. None of the participants fulfilled criteria for psychotic disorders, secondary to a medical condition, as identified in Diagnostic and Statistical Manual of Mental Disorders, Fifth Edition (DSM-5), were under antipsychotic medication or had a history of psychotic illness.

The Questionnaire for Impulsive–Compulsive Disorders in PD-Rating Scale (QUIP-RS) was performed to examine impulsive–compulsive behaviors (ICBs) [including compulsive gambling, buying, sexual behavior and eating] and related disorders (including hobbying, punding and dopamine dysregulation syndrome, DDS). A cut off score of ≥ 10 in QUIP-RS was used to indicate combined ICBs [26]. Subthreshold impulsive symptoms were documented using a cut off score of ≥ 1 in QUIP-RS scale.

### Beads task

JtC bias was measured by a probalistic reasoning scenario, known as the beads task [27]. This computerized task involves showing participants 2 jars of beads in equal but opposite ratios (60 red and 40 blue) and vice versa. Both jars were hidden and subjects were told that individual beads were drawn consecutively from 1 jar. The task of the participant was to decide from which jar beads were being drawn. Two key outcome variables from this procedure are: (a) the number of beads drawn before a decision is made (“draws to decision”, DTD) and (b) the proportion of “extreme responders”. This group involves individuals who make a decision on the basis of 2 or fewer beads (two-bead extreme responders), which indicates the presence of a marked JtC bias. For exploratory purposes, a variable of “extreme responding” based on only one bead to make a decision (single-bead extreme responders) was also computed and compared in PD patients and healthy individuals.

In the current study, the computerized 60:40 version of beads task was performed. In various manipulations of the task, different stimulus pairs are substituted for the two bead colors, or the ratios of the two types of stimuli are changed. The selection of the harder version of beads task was made to achieve more sensitive discrimination of differences between the two study groups [6].

Patients with PD group were categorized in JtC and nJtC subgroups, depending on their performance in beads task. JtC group involved two-bead extreme responders, while nJtC category consisted of patients without early decision on beads task (prediction based on > 2 beads).

### Statistical analysis

Statistical analyses were performed using the statistical software programs IBM SPSS, version 25.0, (USA). Normality of data was graphically explored using Q–Q plots. Categorical variables were summarized as absolute numbers and percentages. Continuous variables were presented as mean ± statistical deviation (SD). DTD and total scores in motor and non-motor scales were treated as continuous variables. The presence of JtC bias, mild cognitive impairment, frontal dysfunction, depressive and psychotic features, ICBs and subthreshold impulsive symptoms were computed as categorical variables.

In the primary analysis, DTD, JtC bias and demographic characteristics (age and sex) were compared between patients with PD and healthy individuals using the chi-square (*χ*^2^) test and Mann–Whitney non-parametric test for not normally distributed variables, as appropriate. Post-hoc *p* values were corrected for multiple comparisons (Bonferonni).

Subsequently, bivariate comparisons between JtC and nJtC subjects were performed using non-parametric Mann–Whitney test (for not normally distributed continuous variables) and the chi-square test (for categorical variables). The level of statistical significance was set at *p* ≤ 0.05.

To further investigate the presence of even subtle cognitive deficits in JtC patients, the individual items of MoCA and FAB scores (either treated as continuous or categorical variables) were compared between JtC and nJtC groups (Supplementary material).

## Results

### JtC bias in patients with PD and healthy individuals

Patients with PD were younger compared to healthy individuals, while there was a male predominance in the PD group, as illustrated in Table 1.

**Table 1:** Demographic characteristics and performance in beads task

	Patients with PD *N* = 68	Healthy subjects *N* = 70	MW u value, *χ*^2^	*p* value
Age mean ± SD	63.2 ± 11.9	67.9 ± 7.9	2099.5	0.050
Male sex *N*, %	47 (67)	27 (37)	13.5	< 0.001
DTD mean ± SD	5.8 ± 4.6	5.6 ± 3.5	2206.0	0.456
Two-bead ER *N*, %	22 (32)	6 (9)	12.1	0.001
Single-bead ER *N*, %	13 (19)	2 (3)	9.4	0.002

*DTD* Draws-to-decision, *ER* extreme responders, *MW* Mann–Whitney non-parametric test, *PD* Parkinson’s disease, *SD* standard deviation

**Fig. 1:**
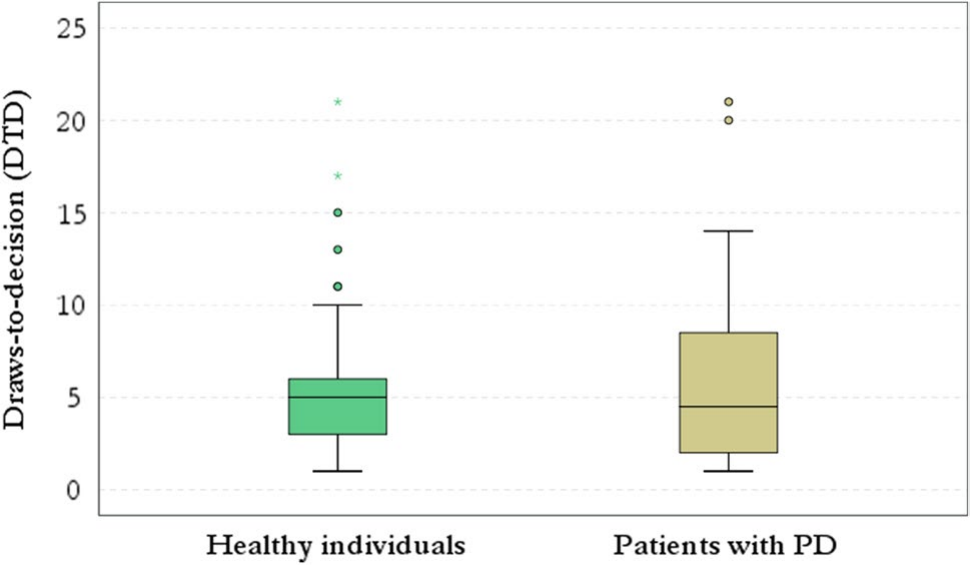
This figure illustrates the distribution of DTD in healthy controls and patients with PD. Compared to healthy individuals, in the PD group, a wide range of DTD was noticed, even though the median DTD were similar in the two groups. DTD: draws-to-decision; PD: Parkinson’s disease

**Fig. 2:**
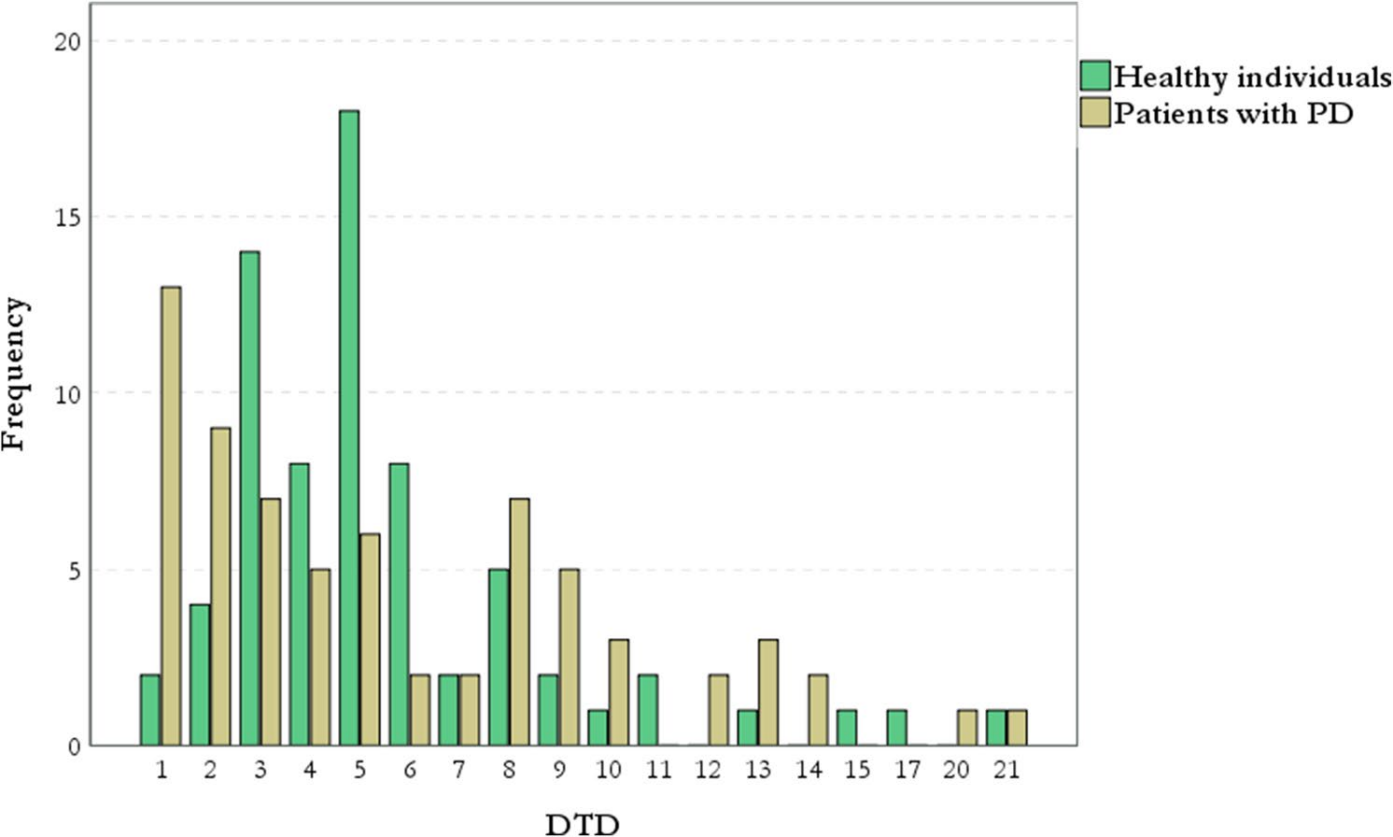
In PD group there is a right-skewed distribution of DTD, compared to healthy subjects. DTD: draws-to-decision; PD: Parkinson’s disease

The median and mean scores of DTD were similar in the two groups (Fig. 1), yet the prevalence of JtC bias was significantly higher in the PD, compared to the control group (Table 1). This difference is further demonstrated by the right-skewed distribution of the DTD in PD patients, compared to a rather bell-shaped distribution in healthy subjects (Fig. 2).

The prevalence of JtC bias in PD group was significantly higher, compared to the control group (32% compared to 9%, respectively). After performing logistic regression analysis, PD patients were six times more likely to manifest JtC compared to healthy controls, after adjusting for age and sex (adjusted ORs: 6.1, 95% CI 2.1–17.6, *p* = 0.001).

Even in the case of single-bead extreme responders, the frequency of JtC bias remained higher in the PD group (Table 1). PD patients were approximately 9 times more likely to develop JtC bias compared to healthy controls, after adjusting for clinical covariates [adjusted ORs: 8.7, 95% CI 1.8–42.8, *p* = 0.008].

### Clinical characteristics of extreme responders in early PD

Age, sex and education status did not differ between the JtC and nJtC groups. (Table 2). Motor performance (MDS-UPDRS III, HY and SE-ADL) was similar between the two groups. Compared to nJtC, JtC subjects had slightly shorter duration of disease (*p* = 0.043). There was a predominance of tremor-dominant subtype in the JTC group (*p* = 0.013).

**Table 2:** Clinical characteristics in JtC and nJtC groups

	JtC group N = 22	nJtC group N = 46	MW u value, χ^2^	*p* value
Demographics				
Age Mean ± SD	63 ± 10.2	63 ± 12.7	480.0	0.733
Male sex * N*, %	13 (59)	32 (70)	0.7	0.393
Education, years Mean ± SD	13.5 ± 4.8	14.7 ± 3.8	384.5	0.223
PD-associated features				
Age of onset Mean ± SD	61.4 ± 10.3	60.9 ± 12.8	488.5	0.818
Duration of disease Mean ± SD	1.5 ± 0.6	1.9 ± 0.8	362.5	0.043
HY Mean ± SD	1.8 ± 0.4	1.7 ± 0.4	483	0.688
SE-ADL Mean ± SD	94.1 ± 5.9	93.5 ± 6.1	450.5	0.725
MDS-UPDRS III Mean ± SD	20.3 ± 7.2	21.5 ± 6.4	424.0	0.504
Tremor subtype * N*, %	7 (32)	3 (7)	8.7	0.015
Medication				
LEDD, mg Mean ± SD	263.1 ± 200.9	226.8 ± 179.0	457.5	0.523
L-dopa * N*, %	12 (55)	23 (50)	0.1	0.726
Dopaminergic agonists *N*, %	11 (50)	20(44)	0.3	0.613
MAO inhibitors *N*, %	6 (27)	9 (20)	0.5	0.473
Non-motor features				
MDS-UPDRS I Mean ± SD	7.9 ± 5.3	5.6 ± 3.0	354.5	0.161
MDS-UPDRS II Mean ± SD	5.0 ± 4.5	6.6 ± 3.8	321.5	0.062
RBDQ Mean ± SD	4.8 ± 2.4	3.9 ± 2.6	371.5	0.096
Sniffin’ Test Mean ± SD	6.5 ± 2.6	6.4 ± 2.6	452.0	0.877

*HY* Hoehn and Yahr scale, *JtC* patients that jumped to conclusions, *LEDD* levodopa equivalent daily dose, *MAO* monoamine oxidase, *MDS-UPDRS* Movement Disorder Society Unified Parkinson’s Disease Rating Scale, *MW* Mann–Whitney non-parametric test, *nJtC* patients that did not jump to conclusions *PD* Parkinson’s disease, *RBDQ* REM sleep behavior disorder screening questionnaire, *SD* Standard Deviation, *SE-ADL* Schwab and England Activities of Daily Living scale

9% (2/22) of the JtC-PD group did not receive any medication at the time of evaluation. No difference was detected in total LEDD and medication strategies between the two groups (Table 2). In both groups, the majority of subjects received levodopa and dopamine receptor agonists, followed by MAO inhibitors and amantadine [JtC group: 55% (12/22), 50% (11/22), 27% (6/22), 5% (1/22); nJtC group: 50% (23/46), 44% (20/46), 20% (9/46), 2% (1/46), *p* = 0.799, *p* = 0.795, *p* = 0.538, *p* = 1.000]. No PD extreme-responder was on COMT inhibitors, in contrast to 5% (1/22) of JtC group (*p* − 0.324).

Non-motor symptoms including olfactory impairment and RBD did not differ between study groups (Table 2).

### JtC bias and neuropsychiatric symptoms in early PD

Global cognitive testing and frontal assessment was similar in JtC and nJtC groups, although there was a trend for an inhibitory control (Go–No-Go) and attention deficit in JtC subjects (Supplementary material).

JtC patients had slightly higher GDS scores and higher prevalence of depressive features, compared to nJtC group, but this difference was not significant (Table 3). Although 16% (11 out of 70) of patients with PD were under antidepressant treatment, no predominance of antidepressant treatment was noted in JtC group (*χ*^2^ = 1.0, *df* = 1, *p* = 0.316).

**Table 3:** Neuropsychiatric features in JtC and nJtC groups

	JtC group *N* = 22	nJtC group *N* = 46	MW u value, *χ*^2^	*p* value
MoCA mean ± SD	25.9 ± 2.3	25.7 ± 3.0	505.0	0.989
Mild cognitive impairment *N*, %	11 (50)	20 (44)	0.3	0.613
FAB mean ± SD	15.9 ± 1.9	15.9 ± 2.7	435.5	0.345
Frontal function *N*, %	2 (9)	6 (13)	0.2	0.636
GDS mean ± SD	4.2 ± 4.1	2.5 ± 2.9	390.5	0.125
Depressive features *N*, %	8 (36)	8 (17)	3.0	0.084
10PDQ mean ± SD	3.6 ± 5.0	2.0 ± 4.1	423.0	0.184
Psychotic features *N*, %	9 (41)	12 (26)	1.5	0.216
Apathy item in MDS-UPDRS I mean ± SD	0.2 ± 0.7	0.1 ± 0.3	439.0	0.954
Apathy *N*, %	2 (9)	4 (9)	0.0	1.000
QUIP-RSmean ± SD	2.3 ± 4.7	0.8 ± 2.9	369.5	0.007
ICBs *N*, %	2 (9)	2 (4)	0.6	0.590
Subthreshold ICBs *N*, %	8 (36)	4 (9)	7.8	0.005

*10PDQ* 10-question PD-specific psychosis severity scale, *FAB* Frontal Assessment Battery, *GDS* Geriatric Depression Scale, ICBs Impulsive–Compulsive Behaviors, *JtC* patients that jumped to conclusions, *nJtC* patients that did not jump to conclusions, *MDS-UPDRS* Movement Disorder Society Unified Parkinson’s Disease Rating Scale, *MoCA* Montreal Cognitive Assessment, *MW* Mann–Whitney non-parametric test, *QUIP* Questionnaire for Impulsive–Compulsive Disorders in PD-Rating Scale, *SD* Standard Deviation

The presence of psychotic features reached 33% in early PD population (23/70), compared to 3% in the control group (2/74) (*p* < 0.001). The majority of PD patients presented minor phenomena (illusions and passage hallucinations), but auditory and olfactory disturbances were also noted in 9% and 6%, respectively, of early PD group. Delusional ideation was rare, occurring in 6% of patients and involved mainly persecution. None of them fulfilled criteria for primary psychotic disorders or received antipsychotic medication. The interplay between psychotic symptoms and JtC bias was explored. The mean 10PDQ scores were higher in JtC patients, compared to nJtC, but this difference did not reach statistical significance (Table 3). 9 out of 22 (41%) of JtC patients reported psychotic features including perceptual abnormalities (minor, visual, auditory and olfactory hallucinations), but no delusional ideation.

**Fig. 3:**
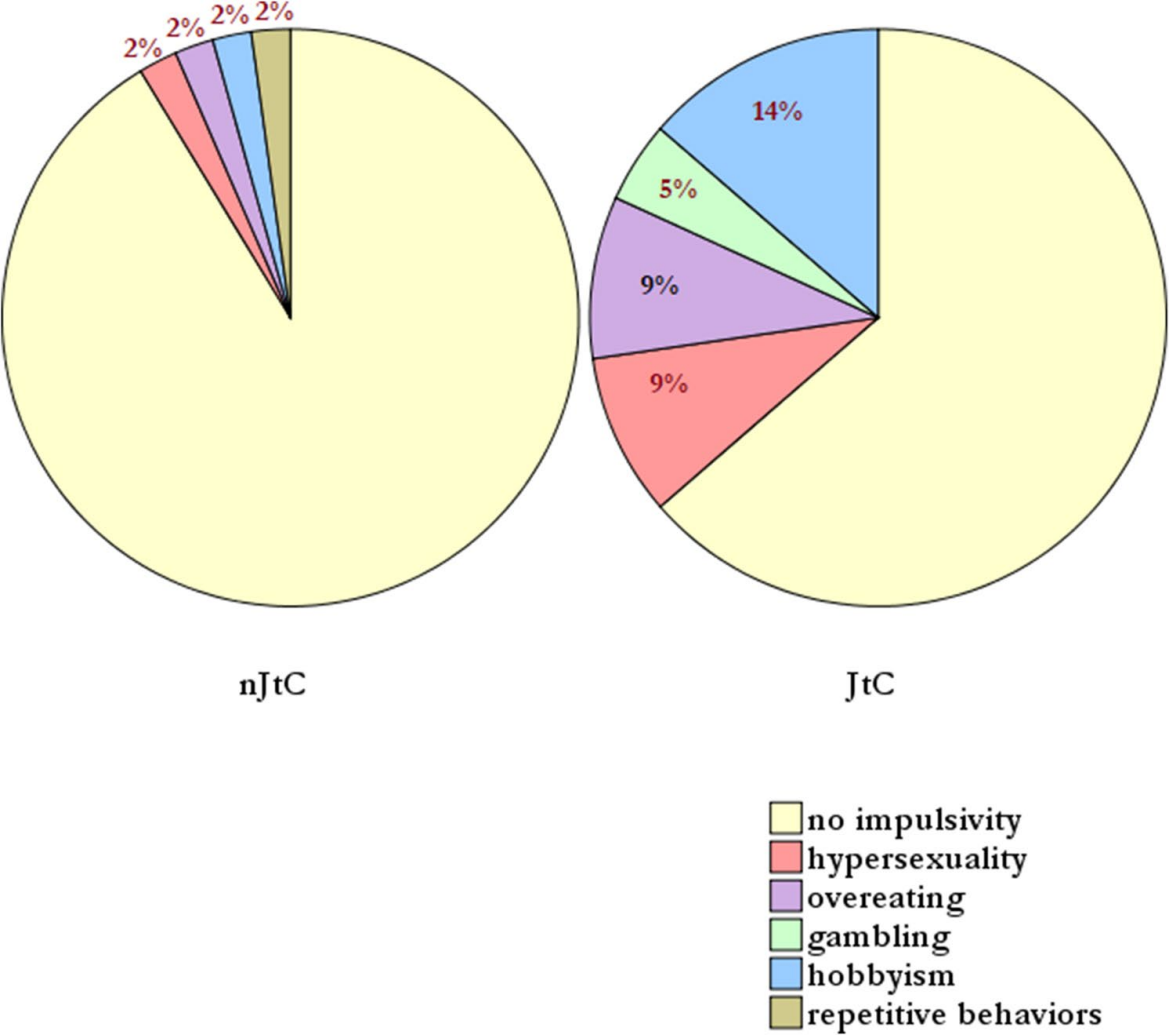
The prevalence of subthreshold impulsive behaviors in JtC and nJtC patients is demonstrated in the current pie chart. Hobbyism followed by overeating, hypersexuality and gambling were the most commonly reported symptoms among JtC patients. In nJtC group, repetitive behaviors, hobbyism, overeating and hypersexuality were equally noticed. JtC: patients that jumped to conclusions, nJtC: patients that did not jump to conclusions

**Fig. 4:**
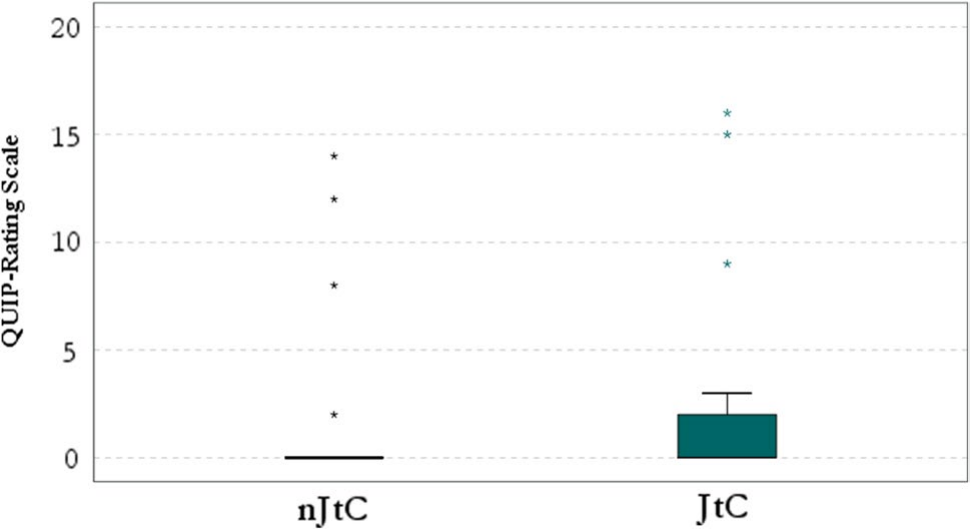
This figure illustrates the distribution of QUIP-RS scores in JtC and nJtC groups. Compared to nJtC patients, in JtC group, the range of QUIP-RS scores was importantly broader, even though the median scores were similar in the two groups. JtC: patients that jumped to conclusions, nJtC: patients that did not jump to conclusions, PD: Parkinson’s disease, QUIP-RS: Questionnaire for Impulsive–Compulsive Disorders in PD-Rating Scale

Figure 2 corresponds to the distribution of DTD in patients with PD and healthy controls. Figure 4 corresponds to the boxplot of QUIP scores in JtC versus nJtC groups and is indicated as (Boxplot). The range and mean scores in QUIP-RS were significantly higher in the JtC group, compared to nJtC individuals [(JTC group: range: 0–16), (nJtC group: range: 0–12] (Boxplot). The prevalence of subthreshold impulsive behaviors was four times higher in JtC patients, compared to nJtC (Table 3). Hobbyism followed by overeating, hypersexuality and gambling were the most commonly reported symptoms among JtC patients. In the nJtC group, repetitive behaviors were present (Fig. 3).

In terms of loss of motivation to act, no association was detected between JtC bias and apathetic symptoms (p > 0.05) (Fig. 4).

## Discussion

This study identified high prevalence of JtC reasoning bias in early PD, compared to age-matched controls. Patients who jumped to conclusions had shorter duration of PD, more tremor-dominant PD subtype and higher scores in ICB scales. No association was detected between JtC bias and the presence of psychotic features. Even though global cognition and frontal function were relatively intact, a trend towards attention and inhibition control deficit was noticed in JtC patients with PD.

The results of the current study revealed a ninefold risk of JtC tendency in early PD. This finding is in accordance with recent literature [28], as even untreated, de novo PD patients had poor ability to gather information [9]. Even though data-gathering bias has been noted in 20% of non-clinical general population [29], in our study, only 9% of healthy individuals jumped to conclusions, while none of them reported any kind of psychotic feature. However, there is a significant discrepancy in the demographic characteristics including age, sex and educational level of the non-clinical “control” group that has been investigated in several studies of primary psychotic samples, compared to our research, which might explain the lower-than-expected rate of JtC bias in the current healthy population. Given that this probalistic reasoning bias has been thought to underpin the formation and maintenance of delusions in individuals with schizophrenia [2, 3], an association between JtC bias and psychotic experiences was anticipated in both groups, yet not observed. In fact, in our early PD population, none of the subjects with JtC tendency reported delusional ideation, but some had perceptual abnormalities, mainly of minor or visual nature. This lack of association between JtC bias and delusions might be attributed to the nature of psychotic phenomena at this early stage of the disease, among which delusions are not prominent. Nonetheless, Edelstyn et al. (2014) first noticed abnormal reasoning in patients with PD and visual hallucinations that could be associated with source attribution errors, where self-generated images were misattributed to an external source [30]. Yet, these findings were based on small PD samples in moderate stages of disease. Our findings are in accordance with the Bristow et al. (2014) study that did not identify differences in hallucinating groups regarding the number of beads drawn or the number of extreme responders on the beads task [31].

Nonetheless, the role of JtC bias in psychotic experiences remains controversial. On one hand, several studies have observed that this reasoning bias in schizophrenia was unrelated to the presence or severity of delusions, suggesting that it may relate more generally to a diagnosis of schizophrenia, while others have reported specificity to delusions in psychosis [32–34]. On the other hand, no correlation has been observed between JtC reasoning bias and delusional ideation in other psychotic samples [3, 35]. Due to this controversy, Lunt and colleagues (2012) have suggested that JtC bias could be an epiphenomenal effect of broader cognitive deficits in schizophrenia and conclude that it might not be a cognitive bias, but a cognitive deficit [36]. In fact, reports have shown that executive measures of working memory could be involved in completing beads task [37, 38], while a left prefrontal involvement has been described in marked JtC tendency [36]. Since, in our study, no association was detected between psychotic features and JtC and a trend towards attention deficit was noted in PD patients who jumped to conclusions, the beads task might reveal subtle prefrontal cognitive dysfunction in early stages of PD that could not be detected by clinical instruments, such as MoCA or FAB scales. Further delineation of the cognitive components that contribute to JtC responses would help develop an understanding of the neurocognitive basis of this phenomenon.

Another key finding of our study was that extreme PD responders had higher QUIP-RS scores, higher prevalence of subthreshold ICBs and showed a mild (non-significant) decrease in inhibitory control. Apart from cognitive bias (or deficit), the beads task has been characterized as a measure of reflection impulsivity, which was first introduced by the matching familiar figures test [39, 40]. Increased reflection impulsivity has been observed in recreational cannabis users [41], individuals with opiate or amphetamine dependence [42] and in PD patients with clinical ICBs [10]. Djamshidian et al. (2012) proposed that, in the latter case, reflection impulsivity could be associated with the initiation of dopaminergic medication, leading possibly to excessive dopamine levels in ventral striatum, due to uneven pattern of dopamine loss, particularly affecting the dorsal striatum [10]. Nevertheless, this could not explain the presence of JtC bias in untreated PD [9], as well as the fact that in our sample, therapeutic strategies and total LEDD were not different between JtC and nJtC groups. Alternatively, the presence of a fourfold prevalence of mild ICBs in JtC patients, could be based on the dysfunction of key regions in prefrontal cortex. Hypoactivity in orbitofrontal cortex and rostral cingulate zone, regions implicated in impulse control, has been found in PD pathological gamblers [43], while these areas have also been implicated in the beads task [36]. Furthermore, premorbid characteristics, such as elevated baseline impulsivity, and disease-related factors, for instance executive dysfunction, play important roles in the presence of JtC reasoning bias in PD patients with ICBs [44, 45]. In the current study, inhibitory control deficits, but not executive dysfunction, failed marginally to associate with JtC bias. Therefore, it is possible that JtC tendency measures an aspect of impulsivity, by unmasking prefrontal deficits, mainly of an attentional and inhibitory control nature, in PD patients with intact cognition during the early stages of the disease. Additional prospective studies would be necessary to explore further the underlying neuropsychiatric mechanisms that modulate the correlation of reflection impulsivity with ICBs in early PD.

Furthermore, motor performance was similar in JtC and nJtC groups of the current analysis. Shorter duration of disease and tremor-dominant phenotype were more prevalent in patients that jumped to conclusions. In PD, tremor-related neural activity has been demonstrated both in the cerebello-thalamo-cortical circuit, and in basal ganglia and related receiving areas of the thalamus [46, 47]. Furthermore, FDG–PET scans have illustrated an oscillatory network that correlates with PD-tremor and included activity in the dentate nucleus, rostral parts of the cerebellum, the putamen and the motor cortex [48]. Interestingly, Schmahmann (1991) suggested that cerebellum regulates the speed, consistency and appropriateness of cognitive processes [49], while an increasing amount of neuroimaging data has illustrated co-activation of the cerebellum with the prefrontal cortex and the temporo-parietal cortex in various types of mental tasks, such as verbal fluency task, card sorting test, Stroop test and Tower of London task [50]. In an evidence-accumulation-based decision-making task, a convergence of task-relevant information onto Purkinje cells suggested that cerebellar activity could play an important role in working memory and decision-making process [51]. Until present, scarce information is available on the association between cerebellar control and the JtC reasoning bias. Our results open up the possibility that implicit cortical and cerebellar mechanisms might be implicated in the probalistic reasoning scenario of beads task.

The current study has several strengths. First, to the best of our knowledge, this is the first research attempt of exploring the association between jumping to conclusion and minor psychotic phenomena in early stages of PD. No association was detected between PD-associated psychosis and the performance in the beads task, which is in accordance with several studies that failed to identify a positive correlation of these entities in other clinical and non-clinical samples. Moreover, tremor-dominant phenotype was more prevalent in PD-extreme responders, despite the similar motor performance and medication therapy between JtC and nJtC groups. The clinical factors that contribute to this correlation are not clear, yet an indication of network dysfunction including cortex and cerebellum has been noted in recent literature. Finally, our findings revealed the bidirectional connection of impulsivity and JtC bias in early PD, as, in the current sample, PD-extreme responders have reported higher impulsivity scores, while literature findings have shown more marked JtC bias in PD-ICBs patients. Mild attention and inhibition control deficits were noted in JtC-PD group, which might indicate the role of prefrontal dysfunction in the association of the two neuropsychiatric entities. More studies are needed to verify these clinical findings.

Our study presents some limitations which need to be acknowledged. First, due to the cross-sectional design of the study, the identification of a temporal and causal relationship between several clinical factors and JtC bias could not be precisely evaluated. In addition, a small proportion of the sample might be contaminated by Lewy body dementia. However, since the diagnosis of PD was based on the MDS diagnostic criteria and none of the included patients had dementia at the time of evaluation, this bias should be negligible. Moreover, information regarding educational attainment or social–economic status was not available, although education years were documented. No important difference regarding educational level was observed between extreme and non-extreme PD responders, which is in accordance with similar data [9]. Another limitation of the current study is related to the origin of the control group. As stated in the “Methods” section, to achieve higher representativeness in terms of age, the control group essentially included spouses of patients with PD who did not present any signs of parkinsonism and had a negative family history of parkinsonism and related disorders. Due to the high motivation and conscientiousness of this control group, compared to the general population, there might be an exaggeration of the observed differences in the performance of beads task between patients and healthy individuals. To overcome this important gap, a strict evaluation of the beads task was held by clinicians and no important overestimation of JtC bias was finally observed. Furthermore, the size of our sample was small. In particular, ICBs were grouped together, regardless of the precise type of impulsivity, such as gambling, hyper sexuality, overeating, and hobbyism. This meant that potential associations with specific symptoms and specific predictors could not be examined due to power concerns. Moreover, since a single trial of beads task was performed, the possibility that participants may have misinterpreted or forgotten the basic principle of the task, which was that beads were only coming from one container rather than both (“miscomprehension” issue) should be stated. Clinicians insisted on a thorough, careful and repetitive explanation of the instructions of the probalistic scenario and encouraged patients to express their hesitations during the performance of the first exploratory assumption to overcome erratic responses. Finally, clinicians were not blind to clinical status (patients-controls); therefore, potential expectational bias should be taken into consideration, in terms of magnifying differences in JtC tendency in patients, compared to controls.

In summary, the present study provides novel evidence that patients with early PD had higher prevalence of JtC reasoning bias and that an important association between impulsivity and JtC tendency exists, regardless of the extent of dopaminergic treatment. Dysfunction of a brain network including prefrontal cortex, striatum and, potentially, cerebellum, which is necessary for decision-making and inhibitory control, could underlie this phenomenon. Further prospective studies could assess these findings and examine whether beads task reflects a possible “red flag” of clinical impulsivity or cognitive impairment in the future stages of PD.

### Supplementary Information

Below is the link to the electronic supplementary material.

Supplementary file1 (DOCX 17 KB)

## Data Availability

Data availability is not currently applicable.

## References

[1] Fine C, Gardener M, Craigie J, Gold I (2007). Hopping, skipping or jumping to conclusions? Clarifying the role of the JTC bias in delusions. Cogn Neuropsychiatry.

[2] Freeman D (2007). Suspicious minds: the psychology of persecutory of delusions. Clin Psychol Rev.

[3] Warman DM, Lysaker PH, Martin JM, Davis L, Haudenschield SL (2007). Jumping to conclusions and the continuum of delusional beliefs. Behav Res Ther.

[4] Clark L, Robbins TW, Ersche KD, Sahakian BJ (2006). Reflection impulsivity in current and former substance users. Biol Psychiatry.

[5] McKenna G, Fox JRE, Haddock G (2014). Investigating the 'jumping to conclusions' bias in people with anorexia nervosa. Eur Eat Disord Rev.

[6] So SH, Siu NY, Wong HL, Chan W, Garety PA (2016). 'Jumping to conclusions' data-gathering bias in psychosis and other psychiatric disorders—two meta-analyses of comparisons between patients and healthy individuals. Clin Psychol Rev.

[7] Aarsland D, Bronnick K, Larsen JP, Tysnes OB, Alves G (2009). Cognitive impairment in incident, untreated Parkinson disease: the Norwegian Park West study Norwegian Park West Study G. Neurology.

[8] Bodi N, Keri S, Nagy H, Moustafa A, Myers CE, Daw N, Dibo G, Takats A, Bereczki D, Gluck MA (2009). Reward-learning and the novelty-seeking personality: a between-and within-subjects study of the effects of dopamine agonists on young Parkinson’s patients. Brain.

[9] de Rezende Costa FH, Averbeck B, O'Sullivan SS, Vincent MB, Rosso AL, Lees AJ, Djamshidian A (2016). Jumping to conclusions in untreated patients with Parkinson's disease. Neuropsychologia.

[10] Djamshidian A, O’Sullivan SS, Sanotsky Y, Sharman S, Matviyenko Y, Foltynie T, Michalczuk R, Aviles-Olmos I, Fedoryshyn L, Doherty KM, Filts Y, Selikhova M, Bowden-Jones H, Joyce E, Lees AJ, Averbeck BB (2012). Decision-making, impulsivity and addictions: do Parkinson’s disease patients jump to conclusions?. Mov Disord.

[11] Robert G, Drapier D, Verin M, Millet B, Azulay JP, Blin O (2009). Cognitive impulsivity in Parkinson’s disease patients: assessment and pathophysiology. Mov Disord.

[12] Kobayakawa M, Koyama S, Mimura M, Kawamura M (2008). Decision making in Parkinson's disease: analysis of behavioral and physiological patterns in the Iowa gambling task. Mov Disord.

[13] Poletti M, Frosini D, Lucetti C, Del Dotto P, Ceravolo R, Bonuccelli U (2010). Decision making in de novo Parkinson's disease. Mov Disord.

[14] Mimura M, Oeda R, Kawamura M (2006). Impaired decision-making in Parkinson's disease. Parkinsonism Relat Disord.

[15] Sáez-Francàs N, Hernández-Vara J, Corominas-Roso M, Alegre J, Jacas C, Casas M (2014). Relationship between poor decision-making process and fatigue perception in Parkinson's disease patients. J Neurol Sci.

[16] Parkin SG, Gregory RP, Scott R, Bain P, Silburn P, Hall B, Boyle R, Joint C, Aziz TZ (2002). Unilateral and bilateral pallidotomy for idiopathic Parkinson's disease: a case series of 115 patients. Mov Disord.

[17] Goetz CG, Tilley BC, Shaftman SR, Stebbins GT, Fahn S, Martinez-Martin P, Poewe W, Sampaio C, Stern MB, Dodel R, Dubois Bruno, Holloway R, Jankovic J, Kulisevsky J, Lang AE, Lees A, Leurgans S, LeWitt PA, Nyenhuis D, Olanow CW, Rascol O, Schrag A, Teresi JA, van Hilten JJ, LaPelle N, Movement Disorder Society UPDRS Revision Task Force (2008). Movement Disorder Society-sponsored revision of the Unified Parkinson's Disease Rating Scale (MDS-UPDRS): scale presentation and clinimetric testing results. Mov Disord.

[18] Hoehn MM, Yahr MD (1967). Parkinsonism: onset, progression and mortality. Neurology.

[19] : Schwab RS and England AC. (1969). Projection techniques for evaluating surgery in Parkinson's Disease. Third Symposium on Parkinson's Disease, Royal College of Surgeons in Edinburgh. E. & S. Livingstone Ltd.

[20] Postuma RB, Arnulf I, Hogl B, Iranzo A, Miyamoto T, Dauvilliers Y, Oertel W, Ju YE, Puligheddu M, Jennum P, Pelletier A, Wolfson C, Leu-Semenescu S, Frauscher B, Miyamoto M, Cochen De Cock V, Unger MM, Stiasny-Kolster K, Fantini ML, Montplaisir JY (2012). A single-question screen for REM sleep behavior disorder: a multicenter validation study. Mov Disord.

[21] Hummel T, Kobal G, Gudziol H, Mackay-Sim A (2007). Normative data for the “Sniffin’ Sticks” including tests of odor identification, odor discrimination, and olfactory thresholds: an upgrade based on a group of more than 3000 subjects. Eur Arch Otorhinolaryngol.

[22] Nasreddine ZS, Phillips NA, Bédirian V, Charbonneau S, Whitehead V, Collin I, Cummings JL, Chertkow H (2005). The Montreal Cognitive Assessment, MoCA: a brief screening tool for mild cognitive impairment. J Am Geriatr Soc.

[23] Dubois B, Slachevsky A, Litvan I, Pillon B (2000). The FAB: a frontal assessment battery at bedside. Neurology.

[24] Yesavage JA, Brink TL, Rose TL, Lum O, Huang V, Adey M, Leirer VO (1982). Development and validation of a geriatric depression screening scale: a preliminary report. J Psychiatr Res.

[25] Ondo WG, Sarfaraz S, Lee M (2015). A novel scale to assess psychosis in patients with Parkinson's disease. J Clin Mov Disord.

[26] Weintraub D, Mamikonyan E, Papay K, Shea JA, Xie SX, Siderowf A (2012). Questionnaire for impulsive-compulsive disorders in Parkinson’s disease-rating scale. Mov Disord.

[27] Phillips LD, Edwards W (1966). Conservatism in a simple probability inference task. J Exp Psychol.

[28] Djamshidian A, O'Sullivan SS, Tomassini FT, Limousin P, Aviles-Olmos I, Warner TT, Lees AJ, Averbeck BB (2014). In a rush to decide: deep brain stimulation and dopamine agonist therapy in Parkinson's disease. J Parkinsons Dis.

[29] Freeman D, Pugh K, Garety P (2008). Jumping to conclusions and paranoid ideation in the general population. Schizophr Res.

[30] Edelstyn NMJ, Drakeford JL, Ellis SJ (2014). Visual hallucinations in Parkinson's Disease: a hierarchy of impairments involving perception, source monitoring and reasoning. Austin J Psychiatry Behav Sci.

[31] Bristow E, Tabraham P, Smedley N, Ward T, Peters E (2014). Jumping to perceptions and to conclusions: specificity to hallucinations and delusions. Schizophr Res.

[32] Peters ER (2003). Cognitive biases involved in the formation of delusional beliefs. Schizophr Res.

[33] Moritz S, Woodward TS (2005). Jumping to conclusions in delusional and non-delusional schizophrenic patients. Br J Clin Psychol.

[34] Van Dael F, Versmissen D, Janssen I, Myin-Germeys I, van Os J, Krabbendam L (2006). Data gathering: biased in psychosis?. Schizophr Bull.

[35] Xenaki LA, Stefanatou P, Ralli E, Hatzimanolis A, Dimitrakopoulos S, Soldatos RF, Vlachos II, Selakovic M, Foteli S, Kosteletos I, Nianiakas N, Ntigridaki A, Triantafyllou TF, Voulgaraki M, Mantonakis L, Tsapas A, Bozikas VP, Kollias K, Stefanis NC (2022). The relationship between early symptom severity, improvement and remission in first episode psychosis with jumping to conclusions. Schizophr Res.

[36] Lunt L, Bramham J, Morris RG, Bullock PR, Selway RP, Xenitidis K, David AS (2012). Prefrontal cortex dysfunction and 'Jumping to Conclusions': bias or deficit?. J Neuropsychol.

[37] Garety P, Freeman D, Jolley S, Ross K, Waller H, Dunn G (2011). Jumping to conclusions: the psychology of delusional reasoning. Adv Psychiatr Treat.

[38] Buck KD, Warman DM, Huddy V, Lysaker PH (2012). The relationship of metacognition with jumping to conclusions among persons with schizophrenia spectrum disorders. Psychopathology.

[39] Dickman SJ, McCown WG, Johnson JL, Shure MB (1993). Impulsivity and information processing. The impulsive client: theory, research and treatment.

[40] Kagan J (1966). Reflection-impulsivity: the generality and dynamics of conceptual tempo. J Abnorm Psychol.

[41] Clark L, Roiser JP, Robbins TW, Sahakian BJ (2009). Disrupted ‘reflection’ impulsivity in cannabis users but not current or former ecstasy users. J Psychopharmacol.

[42] Djamshidian A, Sanotsky Y, Matviyenko Y, O'Sullivan SS, Sharman S, Selikhova M, Fedoryshyn L, Filts Y, Bearn J, Lees AJ, Averbeck BB (2013). Increased reflection impulsivity in patients with ephedrone-induced Parkinsonism. Addiction.

[43] Potenza MN, Winters KC (2003). The neurobiology of pathological gambling: translating research findings into clinical advances. J Gambl Stud.

[44] Poletti M, Bonuccelli U (2013). From aberrant salience to jumping to conclusions: dopaminergic pathways to delusions in Parkinson disease. J Clin Psychopharmacol.

[45] Vitale C, Santangelo G, Trojano L, Verde F, Rocco M, Grossi D, Barone P (2011). Comparative neuropsychological profile of pathological gambling, hypersexuality, and compulsive eating in Parkinson's disease. Mov Disord.

[46] Hirschmann J, Hartmann CJ, Butz M, Hoogenboom N, Özkurt TE, Elben S, Vesper J, Wojtecki L, Schnitzler A (2013). A direct relationship between oscillatory subthalamic nucleus–cortex coupling and rest tremor in Parkinson’s disease. Brain.

[47] Lenz FA, Kwan HC, Martin RL, Tasker RR, Dostrovsky JO, Lenz YE (1994). Single unit analysis of the human ventral thalamic nuclear group. Tremor-related activity in functionally identified cells. Brain.

[48] Mure H, Hirano S, Tang CC, Isaias IU, Antonini A, Ma Y, Dhawan V, Eidelberg D (2011). Parkinson's disease tremor-related metabolic network: characterization, progression, and treatment effects. Neuroimage.

[49] Schmahmann JD (1991). An emerging concept. The cerebellar contribution to higher function. Arch Neurol.

[50] Ito M (2008). Control of mental activities by internal models in the cerebellum. Nat Rev Neurosci.

[51] Deverett B, Koay SA, Oostland M, Wang SS (2018). Cerebellar involvement in an evidence-accumulation decision-making task. eLife.

