# Supplementary material for: Jumping to conclusions bias, psychosis and impulsivity in early stages of Parkinson’s disease
Source: J Neurol. 2023 Aug 9;270(12):5773–83. doi: 10.1007/s00415-023-11904-x (PMC10632276; doi:10.1007/s00415-023-11904-x)
Supplement: Supplementary file 1 — Supplementary file1 (DOCX 17 KB) [file 415_2023_11904_MOESM1_ESM.docx]

| Supplementary Table: Cognitive and Frontal Examination in JtC and nJtC groups | | | | |
| --- | --- | --- | --- | --- |
|  | JtC group  N=22 | nJtC group  N=46 | MW u, χ^2^ | p-value |
| *MoCA* |  |  |  |  |
| Visuospatial-Executive  mean±SD | 3.8±1.2 | 4.0±1.2 | 432.5 | 0.458 |
| Trail Making Test  N,% | 6 (27) | 11 (25) | 0.0 | 0.842 |
| Cube  N,% | 12 (55) | 20 (46) | 0.5 | 0.486 |
| Clock  N,% | 7 (47) | 8 (18) | 1.6 | 0.213 |
| Naming  mean±SD | 2.8±0.5 | 2.9±0.4 | 451.0 | 0.368 |
| Naming  N,% | 3 (14) | 3 (7) | 0.8 | 0.364 |
| Attention  mean±SD | 5.9±0.7 | 5.6±0.9 | 372.5 | 0.063 |
| Attention  N,% | 1 (5) | 11 (25) | 3.9 | 0.049 |
| Language  mean±SD | 1.8±0.5 | 1.7±0.6 | 442.0 | 0.696 |
| Language  N,% | 4 (19) | 10 (23) | 0.1 | 0.736 |
| Fluency  mean±SD | 0.2±0.4 | 0.3±0.5 | 413.5 | 0.371 |
| Fluency  N,% | 17 (81) | 31 (71) | 0.8 | 0.368 |
| Abstraction  mean±SD | 1.8±0.5 | 1.8±0.4 | 457.5 | 0.916 |
| Abstraction  N,% | 3 (14) | 6 (14) | 0.0 | 0.943 |
| Delayed Recall  mean±SD | 3.3±1.6 | 3.1±1.5 | 411.0 | 0.465 |
| Delayed Recall  N,% | 15 (71) | 36 (82) | 0.9 | 0.341 |
| Orientation  mean±SD | 6.0±0.2 | 6.0±0.0 | 440.0 | 0.148 |
| Orientation  N,% | 1 (5) | 0 (0) | 2.1 | 0.145 |
| *FAB* |  |  |  |  |
| Similarities  mean±SD | 2.9±0.3 | 2.9±0.4 | 465.0 | 1.000 |
| Similarities  N,% | 2 (9) | 4 (9) | 0.0 | 0.978 |
| Lexical fluency  mean±SD | 2.4±0.7 | 2.5±0.7 | 465.0 | 0.652 |
| Lexical fluency  N,% | 11 (50) | 19 (42) | 0.4 | 0.548 |
| Luria  mean±SD | 2.6±0.8 | 2.5±0.9 | 456.0 | 0.485 |
| Luria  N,% | 4 (18) | 12 (27) | 0.6 | 0.444 |
| Conflicting Instructions  mean±SD | 2.7±0.6 | 2.7±0.6 | 492.5 | 0.963 |
| Conflicting Instructions  N,% | 5 (22) | 10 (22) | 0.0 | 0.963 |
| Go-No-Go  mean±SD | 2.1±0.8 | 2.5±0.7 | 375.0 | 0.077 |
| Go-No-Go  N,% | 14 (64) | 18 (40) | 3.3 | 0.069 |
| Prehension Behavior  mean±SD | 3.0±0.0 | 2.9±0.3 | 375.0 | 0.319 |
| Prehension Behavior  N,% | 0 (0) | 2 (4) | 1.0 | 1.000 |

The individual items of MoCA and FAB scores (either treated as continuous or categorical variables) were compared between JtC and nJtC groups. There was a trend of inhibitory control (Go-No-Go) and attention deficit in patients that jumped to conclusions. FAB: Frontal Assessment Battery, JtC: patients that jumped to conclusions, nJtC: patients that did not jump to conclusions, MoCA: Montreal Cognitive Assessment, MW: Mann-Whitney non-parametric test, SD: Standard Deviation.
